# Ibuprofen plasma concentration profile in deliberate ibuprofen overdose with circulatory depression treated with therapeutic plasma exchange: a case report

**DOI:** 10.1186/s40360-017-0187-9

**Published:** 2017-12-12

**Authors:** Stefanie Geith, Bertold Renner, Christian Rabe, Jochen Stenzel, Florian Eyer

**Affiliations:** 1 Department of Clinical Toxicology & Poison Control Centre Munich, Technical University of Munich, School of Medicine, Klinikum rechts der Isar, Ismaninger Str. 22, 81675 Munich, Germany; 2 Institute of Experimental and Clinical Pharmacology and Toxicology, University of Erlangen-Nürnberg, Krankenhausstr. 9, 91054 Erlangen, Germany

**Keywords:** Ibuprofen overdose, Therapeutic plasma exchange, Heart/organ/tissue specific complications of poisoning

## Abstract

**Background:**

Inquiries relating to ibuprofen overdose have more than tripled in the last ten years in our poison control center. Although the vast majority of cases have a benign clinical course, there are few severe or even fatal cases present with refractory circulatory failure.

**Case presentation:**

We describe a case of a 48 year-old male with suicidal mono-ingestion of approximately 72 g ibuprofen. Despite an initial rapid spontaneous drop in the total ibuprofen plasma concentration (IPC) from 550 to 275 mcg/mL within the first 5 h after admission, the patient developed a circulatory failure, refractory to aggressive fluid resuscitation and high doses of vasopressors. Due to ibuprofen’s favorable pharmacokinetics (>95% bound to albumin, low volume of distribution) and in the absence of specific therapeutic alternatives thereby avoiding escalating vasopressor doses, therapeutic plasma exchange (TPE) for extracorporeal elimination of ibuprofen was considered as a therapeutic rescue option.

An improvement of hemodynamics with a significant reduction of vasopressors was observed with TPE-initiation. However, neither the observed IPC-profile nor a pharmacokinetic (PK) simulation provided evidence for a quantitative effective elimination of ibuprofen by TPE. Based on PK-modeling we calculated an overall ibuprofen half-life of 17.2 h for the entire observation period over 5 days.

**Conclusions:**

To our knowledge this is the first report of a severe ibuprofen-mono intoxication treated with TPE and providing serial IPCs over a period of five days, indicating an estimated fivefold overall-elimination half-life of 17.2 h. Despite TPE clinically improved persistent hemodynamic instability, this procedure was neither consistent with the observed IPC-profile nor correlated with a meaningful quantitative elimination of ibuprofen.

**Electronic supplementary material:**

The online version of this article (10.1186/s40360-017-0187-9) contains supplementary material, which is available to authorized users.

## Background

There are numerous reports of acute ibuprofen overdoses, but only few of them deal with potentially life-threatening complications, such as protracted circulatory or renal failure [1–4]. Although fatalities have been described, they account for less than 1 % of cases [5–8].

Coingested drugs, comorbidities or secondary complications likely have contributed more to death rather than ibuprofen itself [6, 8–11]. Ibuprofen plasma concentrations (IPC) were frequently not recorded [8]. Therefore, this is the first report of therapeutic plasma exchange (TPE)-use along with serial pharmacokinetic (PK)-data over a period of 5 days in ibuprofen overdose.

## Case presentation

A 48-year-old man was admitted to Intensive Care Unit (ICU) 3.5 h after suicidal ingestion of 90 tablets ibuprofen - 800 mg per tablet. His long-term medication consisted of Ibuprofen 3 × 800 mg per day for chronic pain along with an inhalative beta-sympathomimetic for allergic asthma. He was preclinically found drowsy but still responsive with an initial Glasgow Coma Score (GCS) of 15, that decreased to 5 at ICU admission. No spontaneous emesis occurred. After recovery, the patient provided written informed consent to publication of this case report.

Vital parameters, physical examination and ECG on admission were normal except for tachycardia (116/min) and respiratory rate (23/min). Increasing drowsiness required endotracheal intubation immediately after ICU admission. Laboratory results for electrolytes, liver and kidney were normal beside a pronounced metabolic acidosis (pH 7.16, pO_2_ 72 mmHg, pCO_2_ 57 mmHg, HCO_3_ 20 mmol/L, base excess −9,2 mmol/L), a creatinine in the upper norm range (1.2 mg/dL [ref. 0.6-1.2 mg/dL]) and a slightly elevation of potassium (5.1 mmol/L [ref. 3.5-5.0 mmol/L]). IPC determined by high pressure liquid chromatography (HPLC) [detection-limit <0.1 mcg/mL, coefficient of variation 14.7%] 4 h after ingestion was 550 mcg/mL [therapeutic range 15-30 mcg/mL]. A urine drug screen (HPLC; TOX.I.S.®, Shimadzu, Europe) largely excluded other therapeutic or illegal drugs, albeit a qualitative screening per HPLC might possibly oversee classes of medications that would be detectable e.g. via LC-MS/MS. Blood serum was tested negative for acetaminophen and salicylates.

A single-dose of 50 g activated charcoal via nasogastric tube and a cumulative dose of 200 mL sodium bicarbonate (8.4%) was administered i.v. to correct acidosis.

The patient became progressively hypotensive, requiring increasing doses of norepinephrine (max. dose before TPE: ~0.3 μg/kg/min) despite fluid resuscitation with 2.5 Liters crystalloids before starting TPE. Extracorporeal ibuprofen elimination by means of TPE was started 9 h after ingestion. A centrifugal plasmapheresis was applied, the catheter was placed in the right jugular vein with a starting blood-flow rate of 79.6 mL/h raised to 103 mL/h at the end of the procedure (duration 1 h 50 min). Citrate was used as anticoagulant and a total plasma volume of 3300 mL was withdrawn. After 1 h of TPE, calciumgluconate 10% was supplemented (90 mL/h) and plasma was replaced with 20 units of fresh frozen plasma (FFP). The estimated amount of eliminated ibuprofen was 990 mg (275 μg/mL*3300 ml).

**Fig. 1:**
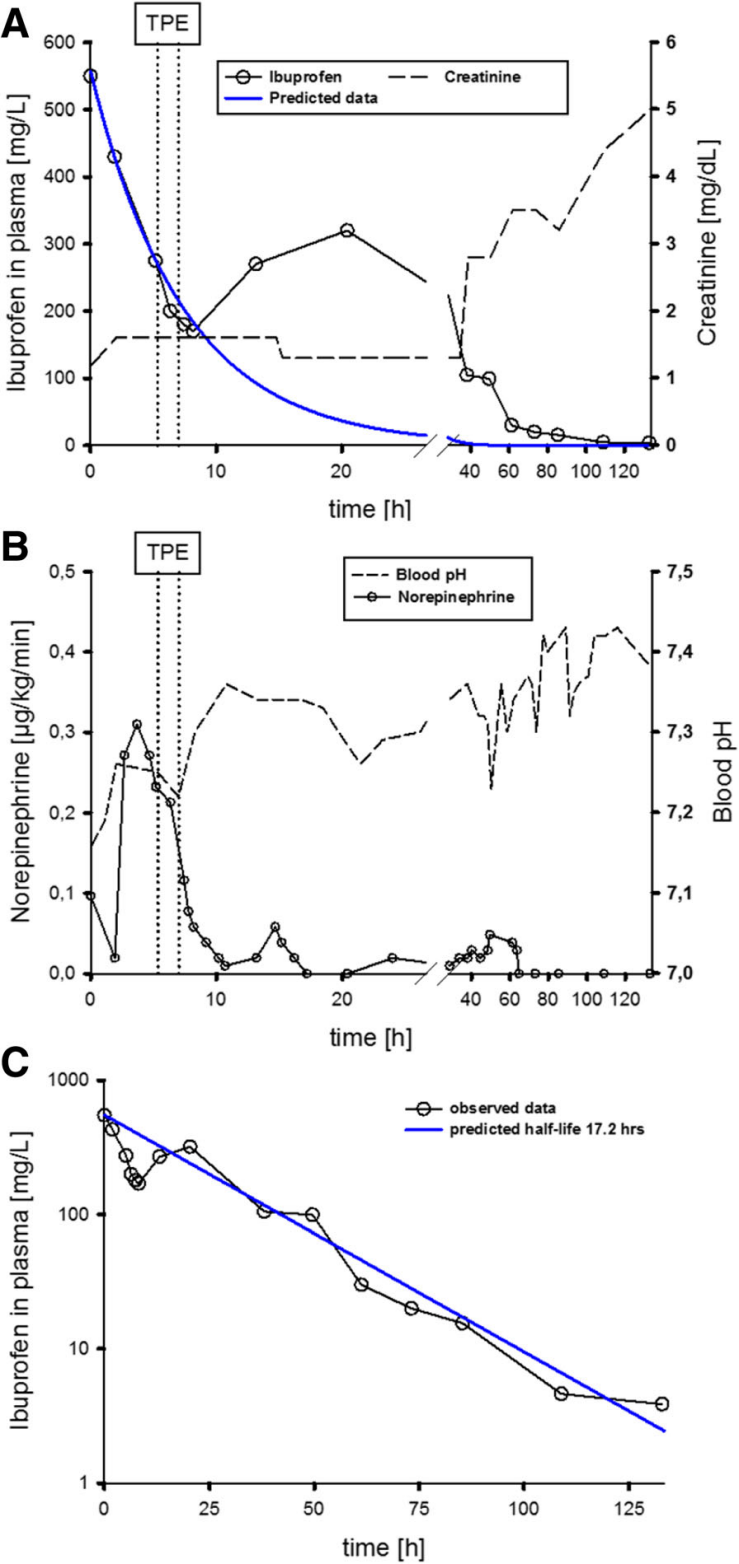
**a** Shown are the measured ibuprofen plasma concentration (IPC) (), creatinine-level (--) and predicted IPC () over time (from ICU admission until day 5). TPE () was applied for about one hour. For simulation of early phase disposition, a one-compartment model with bolus input and 1st order elimination rate was used [C=Co * e(exp(-k * t)); Phoenix WinNonlin 6.3, Pharsight, Mountain View, CA]. This model provided an almost “pseudo-normal” elimination half-life of 5.1 h. **b** Shown are the applied norepinephrine dose () in relation to the blood pH (--) over time. Acidosis improvement was not correlated with the norepinephrine dose (**b**). **c** Measured ibuprofen plasma concentration (IPC) () and predicted IPC () over time (from ICU admission until day 5, linear x-axis). For estimation of the overall elimination rate, a non-compartmental analysis (NCA) was performed. Within the NCA model a linear trapezoidal calculation method with linear interpolation was used. The NCA model provided an estimated overall elimination half-life of 17.2 h

IPC spontaneously decreased from 550 mcg/mL to 275 mcg/mL before TPE with a decline to 180 mcg/mL after TPE (Fig. 1a, Additional file 1). Unexpectedly, IPC increased up to levels of 320 mcg/mL 10 h after TPE and slowly decreased within the following 20 h to 100 mcg/mL. Ibuprofen was completely removed at day 5 (Fig. 1a, Additional file 1).

The patient’s hemodynamic status began to stabilize before TPE, but rapidly improved soon after onset of TPE. Norepinephrine dose was reduced from 0.3 μg/kg/min before, respectively 0.2-0.25 μg/kg/min during to 0.01-0.1 μg/kg/min after TPE [11 h p.i.] (Fig. 1b, Additional file 1).

Acute kidney injury occurred around 40 h after ingestion (creatinine >2.5 mg/dL up to 5 mg/dL at day 5) and required daily hemodialysis for four days and was most probably induced by ibuprofen itself and the associated severe hemodynamic comprise (Fig. 1a, Additional file 1). Other causes were excluded by ultrasonography.

The patient was extubated five days after admission and transferred for further psychiatric treatment on day 12.

## Discussion and conclusions

Contrary to hemodialysis where drugs that are not tightly bound to plasma proteins can effectively been extracted, TPE is more efficient in removing drugs with a high protein binding (i.e. > 80%) and/or a low volume of distribution (e.g. < 0.2 L/kg) [12]. Despite this, TPE is rarely applied or reported as a therapeutic option in the context of intoxication.

In this case of severe ibuprofen mono-intoxication, associated circulatory failure was relatively refractory despite aggressive fluid resuscitation within the first 5 h and norepinephrine application. In the absence of specific therapeutic alternatives, the high rate of protein binding (nearly 99% in therapeutic plasma ranges) and its low volume of distribution (0.11 to 0.19 L/kg), TPE for extracorporeal elimination of ibuprofen was commenced along with serial determination of IPC.

Despite the high dose of ibuprofen ingested, we initially observed an unexpected rapid decline of IPC from 550 mcg/mL to 275 mcg/mL during the first 8 h after admission. The initial decrease reflected a “pseudo-normal” elimination half-life of 5.1 h, but for the entire observation period we estimated a prolonged overall-elimination half-life of 17.2 h (Fig. 1c, Additional file 1), which is more than five times longer than those reported in the literature at therapeutic doses [normal: 1.8 to 3.5 h] [13].

Unexpectedly, this initial spontaneous decrease of IPC, which was only slightly accelerated by TPE, did not correlate with clinical improvement. In contrast, circulatory stabilization with subsequent reduction of norepinephrine dose [from 0.2-0.25 μg/kg/min to 0.01-0.1 μg/kg/min] ensued in close temporal relation to the beginning of TPE regardless of the negative hemodynamic effects of TPE itself and a comparatively small reduction of IPC from 275 mcg/mL to 200 mcg/mL during and 180 mcg/mL after TPE, respectively (Fig. 1a, Additional file 1). Acidosis improvement and norepinephrine dosage were not correlated. The equilibrium of the acidosis was not followed by a norepinephrine dose reduction, which on the contrary had to be increased up to 0.3 μg/kg/min despite rising pH-value. Additionally, during TPE a slightly reducing pH did not result in a norepinephrine dose increase. Norepinephrine was then subsequently reduced during and within the first 10 h after TPE to levels <0.1 μg/kg/min, simultaneously with a further pH stabilization after TPE (Fig. 1b, Additional file 1). The reduction of the norepinephrine-dose prior to TPE was negligible compared with the dose-reduction after TPE and may have been related to the rapid spontaneous decrease of IPC. In line with our findings, several cases in literature in fact describe prolonged circulatory depression, despite declining plasma-levels of ibuprofen [7, 10, 14].

This initially lagged but then sharply decline in IPC has probably been caused by the early distribution phase of the ibuprofen in combination with a delayed absorption due to impaired gastrointestinal tract (GIT)-perfusion as a part of severe hemodynamic comprise, but accompanied by a still persistent elimination.

Reported IPC-correlation with clinical symptoms is quite inconsistent. A relatively mild clinical picture despite high IPCs (e.g. an IPC of 1034 mcg/mL) was associated with a moderate clinical course only with coma and mild metabolic acidosis [11], while another case (IPC of 1050 mcg/mL) was fatal [7]. In other reports, IPC of 704 mcg/mL caused no symptoms [10], while low IPCs (42 mcg/mL and 72 mcg/mL respectively) [11] caused severe or even fatal courses. Reported serious or even fatal overdoses may have also been attributed to complications, such as secondary sepsis or coingested substances [11]. A possible explanation for this discrepancy between IPC and clinical presentation could be the fact that in the vast majority of cases only a single determination of IPC - within the first few hours after ingestion - rather than a concentration-profile was determined. Thus the maximum of IPC either remained undiscovered or reflected the early phase of absorption. We avoided these limitations by repeated blood sampling.

In contrast to the obvious clinical improvement during TPE, the decrease in IPC was comparatively small during/after TPE compared to the rather large initial and spontaneous decline in IPC before TPE was instituted. Unexpectedly, after termination of TPE, IPC re-increased from 170 mcg/mL 8 h after admission (i.e. 12 h p.i.) to a maximum of 320 mcg/mL 20 h after admission (i.e. 24 h p.i.), which deserve a further comment.

Redistribution of ibuprofen from the periphery caused by an increased protein binding capacity following TPE with FFP as a substitute, thereby supplying additional protein – mostly albumin - for binding leading to a rise in total IPC. Independent of this the pH influences the albumin-binding-capacity to drugs as shown for diclofenac [15]. In the acidotic environment, the binding-capacity is reduced, therefore the ratio of free to bound ligand increases with the stabilization of the pH [16]. Unfortunately, only total IPCs were measured in our case, so that no differentiation was possible between the fraction of protein-bound and freely circulating ibuprofen. Due to saturation in protein binding in massive overdoses, a presumably higher proportion of non-protein-bound ibuprofen is to be expected. In rats, the non-protein-bound proportion of ibuprofen (5.5%) was relatively stable up to concentrations of 90 mg/L, but increased concentration-dependent at higher doses [17]. Thus, earliest TPE onset for drug removal plus determination of both free and protein bound ibuprofen seems to be desirable in future cases to better evaluate whether e.g. albumin-based dialysis-techniques or even TPE may – if at all - be beneficial.

TPE along with hemodynamic stabilization may have improved the perfusion of the GIT in our patient, thereby increasing the absorption of ibuprofen. A quantitative eliminative effect of TPE may have even prevented a more pronounced increase of IPC than that observed. Ibuprofen eliminated by TPE was estimated by sample calculation based on a plasma turnover of 3300 mL, resulting in an ibuprofen amount of 990 mg, about 1/100 of the ingested dose, which is clinically negligible. To achieve a clinically relevant elimination, repeated TPE or exchange of a larger plasma volume may be considered in cases with prolonged hemodynamic instability.

Finally, additional hemodialysis due to persistent acute renal failure in the later course may have contributed to the overall clinical improvement in this case although it is unlikely that hemodialysis has any relevant effect on ibuprofen clearance.

The exact pathophysiology of ibuprofen-induced shock is largely unknown and remains speculative. Metabolic acidosis in ibuprofen overdose is believed to be caused by its metabolism to weak acids and may affect cardiovascular function, decrease cardiac output and vascular tone, and contribute to profound hypotension [18, 19]. Furthermore, drug-induced Takotsubo cardiomyopathy has been described in a combined ibuprofen and diphenhydramine-overdose [20], however, echocardiography in our own case does not point to such a condition. Finally, functional adrenal insufficiency with refractory hypotension in a combined naproxen and ibuprofen overdose – responding to hydrocortisone – was another issue discussed in the literature. Prostaglandins, synthesized by cyclooxygenases (COX-1 and COX-2 isoenzymes) - are present in brain structures and are involved in activation of the hypothalamic-pituitary-adrenal axis [21]. This may be blunted by NSAID-induced inhibition of COX-1 and COX-2, such as naproxen or ibuprofen [22]. We cannot rule out such a condition in our patient as we neither determined cortisol level nor we administered hydrocortisone therapeutically. Ultimately, ibuprofens known hyperosmolality in aqueous solutions of the gut after ingestion of very high doses may have caused dehydration, contributing to hypotension.

To the best of our knowledge we report the first ibuprofen overdose with providing IPC-kinetics over a five-day period. Data indicate an overall elimination half-life of 17.2 h, far longer than reported for therapeutic doses [13]. Neither serial IPC-sampling nor PK-simulation indicate TPE-effectiveness in elimination of ibuprofen in this case. Costs, side effects and limited availability contraindicate TPE, regardless of a direct temporal association with hemodynamic improvement.

However, individual pharmacokinetic profiles, ibuprofen’s protein binding properties in the overdose setting and particularly during and shortly after TPE-initiation require further research. To develop a complete IPC-profile and to evaluate the ibuprofen protein-binding capacities in the overdose setting, blood plasma concentrations of protein-bound and free ibuprofen together with determination of the amount of eliminated ibuprofen in the discarded plasma by TPE should be determined.

## Additional file

Additional file 1: Measured Ibuprofen in plasma (IPC), creatinine and norepinephrine doses, and predicted IPC elimination. Raw Data Set of the measured Ibuprofen in plasma, creatinine and norepinephrine levels and the calculated values for the interpolation of predicted IPC elimination. A - Time course of Ibuprofen plasma concentration measured/predicted and serum-creatinine. B – Norepinephrine-dose and blood pH over time. C – Logarithmic plasma concentration of Ibuprofen (measured/predicted) over time. (XLSX 53 kb)

## Abbreviations

FFP: Fresh frozen plasma
GCS: Glasgow Coma Score
GIT: Gastro intestinal tract
HPLC: High Pressure Liquid Chromtography
ICU: Intensive Care Unit
IPC: Ibuprofen plasma concentration
PK: Pharmacokinetic
TPE: Therapeutic plasma exchange
